# Innovations in Rural Aging: Community-Based Approaches to Support Older Adults

**DOI:** 10.1093/geroni/igaf122.831

**Published:** 2025-12-31

**Authors:** Heather Fuller

## Abstract

Rural communities provide a unique context, posing both challenges and advantages as individuals age. The purpose of this symposium is to showcase innovative community-based approaches to assessing needs and implementing interventions for rural older adults across five distinct U.S. regions. Monaghan-Geernaert used a community-driven approach to examine modalities of caregiving for Native American elders within rural settings in South Dakota, finding that implementing Indigenous healing methods into care environments helps improve patient well-being and caregiver effectiveness. Kopera-Frye examined caregiving needs among rural New Mexico Latinx caregivers and found culturally appropriate, family-based solutions for caregiving despite gaps in access to formal support services. Liu et al. examined challenges faced by rural older Veterans with vision loss in Northern Florida/South Georgia, determining that implementing low-tech solutions by generalist healthcare providers could support aging well in rural communities and sharing initial implementation steps of a low-tech toolkit intervention to foster independent living. Roberts & Chen implemented an intergenerational Active Aging for L.I.F.E. project for promoting shared positive health perspectives among older adults and young adults in rural Oklahoma and found outcomes demonstrating effectiveness for train the trainer programming. Fuller et al. assessed the implementation and effectiveness of the community-driven Aging in Community program for fostering aging in place in rural North Dakota, finding that a responsive and flexible intervention resulted in older adults’ improved independence, community connection, and quality of life. Together, these papers highlight innovative strategies for addressing the diverse needs of aging in rural communities, emphasizing the importance of community-informed approaches. Rural Aging Interest Group Sponsored Symposium

